# Supplementary material for: Serology reflects a decline in the prevalence of trachoma in two regions of The Gambia
Source: Sci Rep. 2017 Nov 8;7:15040. doi: 10.1038/s41598-017-15056-7 (PMC5678181; doi:10.1038/s41598-017-15056-7)
Supplement: Supplementary file 1 — Supplementary Information [file 41598_2017_15056_MOESM1_ESM.doc]

TITLE

Serology reflects a decline in the prevalence of trachoma in two regions of The Gambia

AUTHORS

Stephanie J Migchelsen^1^*, Nuno Sepúlveda^1,2^, Diana L Martin^3^, Gretchen Cooley^3^, Sarah Gwyn^4^, Harry Pickering^1^, Hassan Joof^5^, Pateh Makalo^5^, Robin Bailey^1^, Sarah E. Burr^1,5^, David CW Mabey^1^, Anthony W Solomon^1^†, and Chrissy h Roberts^1^†

1. Clinical Research Department, London School of Hygiene & Tropical Medicine, London, United Kingdom
2. Centro de Estatística e Aplicações, Faculdade de Ciências, Universidade de Lisboa, Lisboa, Portugal
3. Division of Parasitic Diseases and Malaria, Centers for Disease Control and Prevention, Atlanta, Georgia, USA
4. IHRC, Inc., Centers for Disease Control and Prevention, Atlanta, Georgia, USA
5. Disease Control and Elimination Theme, Medical Research Council, The Gambia Unit, Fajara, The Gambia

† Contributed equally

SUPPLEMENTARY INFORMATION

* Corresponding Author Details
Stephanie J. Migchelsen
Tel : +44 (0) 207 927 2419
Email : [stephanie.migchelsen@lshtm.ac.uk](mailto:stephanie.migchelsen@lshtm.ac.uk)

SUPPLEMENTARY INFORMATION

We provide a more detailed description of the prevalence of clinical signs by age and gender in Supplementary Table 1, including prevalence in one-year increments for children 9 years and under, as well as extending the age groups up to 60+ years of age.

Supplementary Table 1. Clinical signs assessed in participants in The Gambia

|  | **Prevalence of clinical signs (%)** | | | | | |
| --- | --- | --- | --- | --- | --- | --- |
|  | **N** | **TF** | **TI** | **TS** | **TT** | **CO** |
| **Overall** | 1832 | 30 (1.6) | 4 (0.2) | 78 (4.3) | 8 (0.4) | 1 (0.1) |
| LRR | 1010 | 18 (1.8) | 4 (0.4) | 55 (5.4) | 7 (0.7) | 1 (0.1) |
| URR | 822 | 12 (1.5) | 0 | 23 (2.8) | 1 (0.1) | 0 |
| Female | 1057 | 10 (0.9) | 3 (0.3) | 52 (4.9) | 5 (0.5) | 1 (0.1) |
| Male | 776 | 20 (2.6) | 1 (0.1) | 26 (3.4) | 3 (0.4) | 0 |
| 1 year old | 65 | 1 (1.5) | 0 | 0 | 0 | 0 |
| 2 years old | 88 | 3 (3.4) | 0 | 2 (2.3) | 0 | 0 |
| 3 years old | 101 | 8 (7.9) | 0 | 1 (1.0) | 0 | 0 |
| 4 years old | 96 | 5 (5.2) | 0 | 1 (1.0) | 0 | 0 |
| 5 years old | 96 | 3 (3.1) | 0 | 1 (1.0) | 0 | 0 |
| 6 years old | 89 | 0 | 0 | 2 (2.2) | 0 | 0 |
| 7 years old | 77 | 2 (2.6) | 1 (1.3) | 0 | 0 | 0 |
| 8 years old | 78 | 1 (1.3) | 0 | 0 | 0 | 0 |
| 9 years old | 52 | 2 (3.8) | 1 (1.9) | 0 | 0 | 0 |
| 10-19 | 412 | 4 (1.0) | 1 (0.2) | 2 (0.5) | 0 | 0 |
| 20-29 | 191 | 0 | 0 | 1 (0.5) | 0 | 0 |
| 30-39 | 152 | 1 (0.7) | 1 (0.7) | 5 (3.3) | 0 | 0 |
| 40-49 | 99 | 0 | 0 | 5 (5.1) | 0 | 0 |
| 50-59 | 95 | 0 | 0 | 15 (15.8) | 2 (2.1) | 0 |
| 60+ | 141 | 0 | 0 | 43 (30.5) | 6 (4.3) | 1 (0.7) |
| 1-9 year olds -LRR | 383 | 14 (3.7) | 2 (0.5) | 1 (0.3) | 0 | 0 |
| 1-9 year olds -URR | 359 | 11 (3.1) | 0 | 6 (1.7) | 0 | 0 |
| ≥10 year olds-LRR | 627 | 4 (0.6) | 2 (0.3) | 54 (8.6) | 7 (1.1) | 1 (0.2) |
| ≥10 year olds-URR | 463 | 1 (0.2) | 0 | 17 (3.7) | 1 (0.2) | 0 |

TF = trachomatous inflammation, follicular; TI = trachomatous inflammation-intense; TS = trachomatous scarring; TT = trachomatous trichiasis; CO = corneal opacity
LRR = Lower River Region; URR = Upper River Region

Similarly, we provide a more detailed description of the seroprevalence of anti-Pgp3 antibodies in LRR and URR by region, gender and age in Supplementary Table 2. We again provide the seroprevalence in one-year increments for children 9 years and under, to facilitate comparison with other seroprevalence studies.

Table 2. Seroprevalence of anti-Pgp3 antibodies in The Gambia by region, gender and age

|  | % seropositive (95% confidence interval) | | | | | | | | | | | |
| --- | --- | --- | --- | --- | --- | --- | --- | --- | --- | --- | --- | --- |
|  | Overall | | | | Lower River Region | | | | Upper River Region | | | |
|  | N | n | % | 95%CI | N | n | % | 95%CI | N | n | % | 95% CI |
| **Overall** | 1868 | 489 | 26.18% | (24.2-28.2) | 1028 | 313 | 30.45% | (27.7-33.4) | 840 | 176 | 20.95% | (18.3-23.9) |
| Female | 1080 | 341 | 31.57% | (28.8-34.5) | 597 | 215 | 36.01% | (32.2-40.0) | 483 | 126 | 26.09% | (22.2-30.2) |
| Male | 788 | 148 | 18.78% | (16.1-21.7) | 431 | 98 | 22.74% | (18.9-27.0) | 357 | 50 | 14.01% | (10.7-18.1) |
| 1 year old | 65 | 2 | 3.08% | (0.5-11.6) | 29 | 0 | 0 | (0-1.9) | 36 | 2 | 5.56% | (1.0-20.0) |
| 2 years old | 88 | 2 | 2.27% | (0.4-8.7) | 33 | 2 | 6.06% | (1.1-21.6) | 55 | 0 | 0 | (0-8.1) |
| 3 years old | 101 | 6 | 5.94% | (2.4-13.0) | 59 | 2 | 3.39% | (0.6-12.7) | 42 | 4 | 9.52% | (3.1-23.5) |
| 4 years old | 96 | 7 | 7.29% | (3.2-14.90 | 46 | 3 | 6.50% | (1.7-18.9) | 50 | 4 | 8.00% | (2.6-20.1) |
| 5 years old | 96 | 6 | 6.25% | (2.6-13.6) | 58 | 4 | 6.90% | (2.2-17.5) | 38 | 2 | 5.26% | (1.0-19.1) |
| 6 years old | 89 | 4 | 4.49% | (1.4-11.7) | 40 | 2 | 5.00% | (0.9-18.2) | 49 | 2 | 4.08% | (0.7-15.1) |
| 7 years old | 77 | 6 | 7.79% | (3.2-16.8) | 42 | 2 | 4.80% | (0.8-17.4) | 35 | 4 | 11.43% | (3.7-27.7) |
| 8 years old | 78 | 6 | 7.69% | (3.2-16.6) | 41 | 3 | 7.30% | (1.9-21.0) | 37 | 3 | 8.11% | (2.1-23.0) |
| 9 years old | 52 | 8 | 15.38% | (7.3-28.6) | 35 | 7 | 20.00% | (9.1-37.5) | 17 | 1 | 5.88% | (0.3-30.8) |
| 1-9 | 742 | 47 | 6.33% | (4.5-8.0) | 383 | 25 | 6.53% | (3.9-8.9) | 359 | 22 | 6.13% | (3.8-8.8) |
| 10-19 | 412 | 65 | 15.78% | (12.2-19.5) | 231 | 40 | 17.32% | (12.8-23.0) | 181 | 24 | 13.26% | (8.8-19.3) |
| 20-29 | 191 | 60 | 31.41% | (25.0-38.6) | 101 | 35 | 34.65% | (25.6-44.8) | 90 | 25 | 27.78% | (19.1-38.4) |
| 30-39 | 152 | 79 | 51.97% | (43.8-60.1) | 79 | 44 | 55.70% | (44.1-66.7) | 73 | 35 | 47.95% | (36.2-59.9) |
| 40-49 | 99 | 65 | 65.66% | (55.4-74.7) | 62 | 47 | 75.81% | (63.0-85.4) | 37 | 18 | 48.65% | (32.2-65.3) |
| 50-59 | 95 | 69 | 72.63% | (62.4-81.0) | 59 | 46 | 77.97% | (64.9-87.3) | 36 | 23 | 63.89% | (46.2-78.7) |
| 60+ | 141 | 104 | 73.76% | (65.6-80.6) | 95 | 75 | 78.95% | (69.1-86.4) | 46 | 29 | 63.04% | (47.5-76.4) |

In our paper, we set the threshold for positivity at the mean of the Gaussian distribution of the seronegative population plus four standard deviations, 0.810 OD_450nm_. Previous studies using the Pgp3 ELISA have commonly used a threshold set as three standard deviations above the mean of the negative population (the 97.5% confidence interval) ^15,20,21^. Supplementary Figure 1 shows the positive and negative populations with both thresholds.


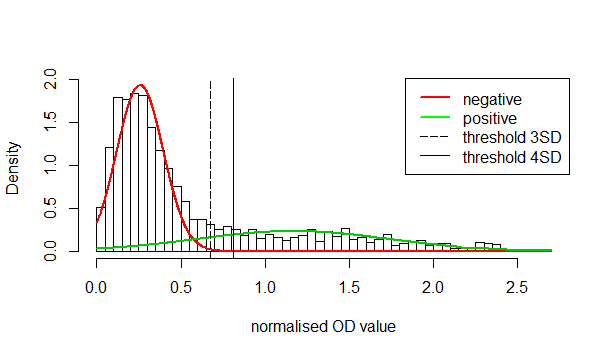


Supplementary Figure 1. Assumed seronegative (red) and assumed seropositive (green) populations as estimated using Finite Mixture Modelling. The dashed line shows the threshold set using the mean of the negative population plus three standard deviations, while the solid line show the more specific threshold set using the mean of the negative population plus four standard deviations.

Below we present the results had we used the typical threshold set using the mean of the Gaussian distribution of the seronegative population plus three standard deviations, 0.673 OD_450nm_. Note that the estimated time change in seroconversion rates does not change.

Supplementary Table 3. Seroprevalence of anti-Pgp3 antibodies by region, gender and age, Lower River Region and Upper River Region, The Gambia, 2014.

|  | **Both regions combined** | | | **Lower River Region** | | | **Upper River Region** | | |
| --- | --- | --- | --- | --- | --- | --- | --- | --- | --- |
|  |  | **Prevalence** | |  | **Prevalence** | |  | **Prevalence** | |
|  | N | % | 95%CI | N | % | 95%CI | N | % | 95% CI |
| **Overall** | 1868 | 26.2 | (24.2-28.2) | 1028 | 30.5 | (27.7-33.4) | 840 | 20.9 | (18.3-23.9) |
| **Gender** |  |  |  |  |  |  |  |  |  |
| Female | 1080 | 31.6 | (28.8-34.5) | 597 | 36.0 | (32.2-40.0) | 483 | 26.1 | (22.2-30.2) |
| Male | 788 | 18.8 | (16.1-21.7) | 431 | 22.7 | (18.9-27.0) | 357 | 14.0 | (10.7-18.1) |
| **Age (years)** |  |  |  |  |  |  |  |  |  |
| 1-9 | 742 | 6.3 | (4.5-8.0) | 383 | 6.5 | (3.9-8.9) | 359 | 6.1 | (3.8-8.8) |
| 10-19 | 412 | 15.8 | (12.2-19.5) | 231 | 17.3 | (12.8-23.0) | 181 | 13.3 | (8.8-19.3) |
| 20-29 | 191 | 31.4 | (25.0-38.6) | 101 | 34.6 | (25.6-44.8) | 90 | 27.8 | (19.1-38.4) |
| 30-39 | 152 | 51.9 | (43.8-60.1) | 79 | 55.7 | (44.1-66.7) | 73 | 47.9 | (36.2-59.9) |
| 40+ | 335 | 71.0 | (65.8-75.8) | 216 | 77.8 | (71.5-83.0) | 119 | 35.2 | (28.6-42.3) |


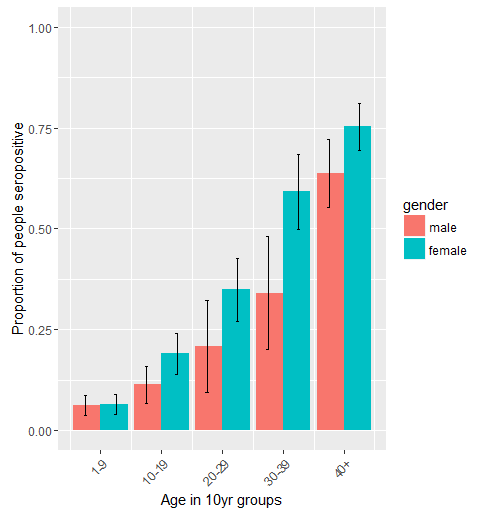


Supplementary Figure 2. Proportion of participants who were seropositive for anti-Pgp3 antibodies using a threshold set at 0.673 OD_450nm_,by age group and region, Lower River Region (LRR) and Upper River Region (URR), The Gambia, 2014. Vertical bars indicate 95% CIs.


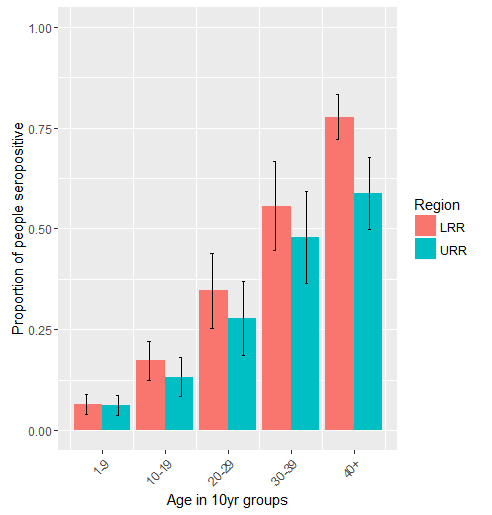


Supplementary Figure 3. Proportion of participants who were seropositive for anti-Pgp3 antibodies using a threshold set at 0.673 OD_450nm_, by age group and gender, Lower River Region and Upper River Region, The Gambia, 2014. Vertical bars indicate 95% confidence intervals.

Supplementary Table 4. Maximum likelihood parameter estimates and the respective 95% confidence intervals (in brackets) for the seroconversion and seroreversion rates (SCR and SRR, respectively) associated data collected from participants in Lower River Region and Upper River Region, The Gambia, 2014. P-values <0.05 are indicative of a change in transmission intensity when comparing two reversible catalytic models, one assuming constant and stable transmission over and another assuming a sudden reduction in transmission intensity somewhere in the past.

|  | **SCR_past_** | **SCR_current_** | **SRR** | **Fold change** | **p-value** |
| --- | --- | --- | --- | --- | --- |
| **Region** |  |  |  |  |  |
| **Lower River Region** | 0.062  (0.038, 0.100) | 0.014  (0.011, 0.018) | 0.006  (0.003,0.014) | 4.4 | <0.001 |
| **Upper River Region** | 0.038  (0.017, 0.082) | 0.011  (0.009, 0.016) | 0.011  (0.003, 0.039) | 3.5 | <0.001 |
